# AI Scribes in Health Care: Balancing Transformative Potential With Responsible Integration

**DOI:** 10.2196/80898

**Published:** 2025-08-01

**Authors:** Tiffany I Leung, Andrew J Coristine, Arriel Benis

**Affiliations:** 1 JMIR Publications, 130 Queens Quay East, Unit 1100, Toronto, ON, M5A 0P6, Canada, 1 4165832040; 2 Department of Internal Medicine (adjunct), Southern Illinois University School of Medicine, Springfield, IL, United States; 3 Division of Cardiology (affiliate), Department of Medicine, McGill University, Montreal, QC, Canada; 4 Department of Digital Medical Technologies, Holon Institute of Technology, Holon, Israel

**Keywords:** ambient AI scribe, digital scribe, AI scribe, virtual scribe, documentation assistant, ambient listening technology, AI assistant, artificial intelligence, clinical documentation, electronic health records, administrative burden

## Abstract

The administrative burden of clinical documentation contributes to health care practitioner burnout and diverts valuable time away from direct patient care. Ambient artificial intelligence (AI) scribes—also called “digital scribes” or “AI scribes”—are emerging as a promising solution, given their potential to automate clinical note generation and reduce clinician workload, and those specifically built on a large language model (LLM) are emerging as technologies for facilitating real-time clinical documentation tasks. This potentially transformative development has a foundation on longer-standing, AI-based transcription software, which uses automated speech recognition and/or natural language processing. Recent studies have highlighted the potential impact of ambient AI scribes on clinician well-being, workflow efficiency, documentation quality, user experience, and patient interaction. So far, limited evidence indicates that ambient AI scribes are associated with reduced clinician burnout, lower cognitive task load, and significant time savings in documentation, particularly in after-hours electronic health record (EHR) work. One consistently reported benefit is the improvement in the patient-physician interaction, as physicians feel more present during a clinical encounter. However, these benefits are counterbalanced by persisting concerns regarding the accuracy, consistency, language use, and style of AI-generated notes. Studies noting errors, omissions, or hallucinations caution that diligent clinician oversight is necessary. The user experience is also heterogeneous, with benefits varying by specialty and individual workflow. Further, there are concerns about ethical and legal issues, algorithmic bias, the potential for long-term “cognitive debt” from overreliance on AI, and even the potential loss of physician autonomy. Additional pragmatic concerns include security, privacy, integration, interoperability, user acceptance and training, and the cost-effectiveness of adoption at scale. Finally, limited studies describe adoption or evaluation of these technologies by nonphysician clinicians and health professionals. Although ambient AI scribes and AI-driven documentation technologies are promising as potentially practice-changing tools, there are many questions remaining. Key issues persist, including responsible deployment with the goal of ensuring that ambient AI scribes produce clinical documentation that supports more efficient, equitable, and patient-centered care. To advance our collective understanding and address key issues, *JMIR Medical Informatics* is launching a call for papers for a new section on “Ambient AI Scribes and AI-Driven Documentation Technologies.” As editors, we look forward to the opportunity to advance the science and understanding of these fields through publishing high-quality and rigorous scholarly work in this new section of *JMIR Medical Informatics*.

## Introduction

Administrative burdens associated with widespread electronic health record (EHR) adoption are well documented, as are the associated clinician burnout and negative consequences for direct patient care and the patient experience. In response, ambient artificial intelligence (AI) scribes have emerged as promising transformative technologies. These technologies aim to listen to patient-practitioner conversations during clinic visits or other synchronous encounters; then, they generate clinical notes for health care practitioner review, revision, and approval. AI scribes are still in the early stages of adoption and evaluation; however, they are already seen operationally as powerful tools that could combat administrative burdens and clinician burnout. Accelerating efforts to install AI scribes in clinical practices are taking place, with a backdrop of long-standing efforts to automate all aspects of documentation workload, which typically includes clinical note generation and other forms of administrative burden, such as preparation of computerized order entry, prior authorization forms, and medical assessments according to structured requirements, as well as coding and billing [1]. In this editorial, we describe our interpretations of the current landscape of ambient AI scribe technology and opportunities for further research and publication, as a part of the *JMIR Medical Informatics* call for papers on “Ambient AI Scribes and AI-Driven Documentation Technologies” [2].

## Hype and Hope of Ambient AI Scribes

**Figure 1.**
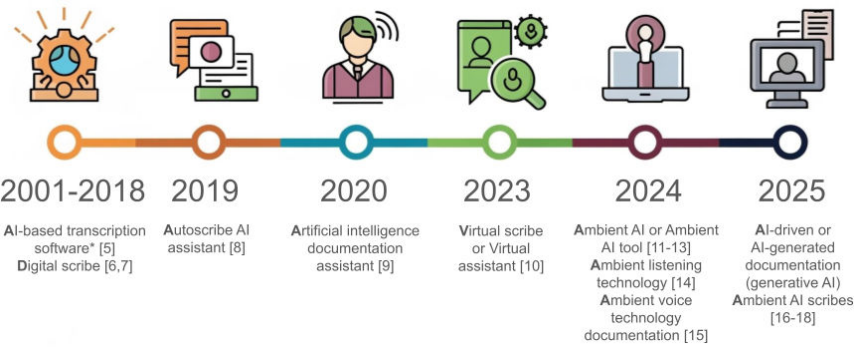
Terminology used for artificial intelligence (AI)–based documentation technologies, based on published literature over the past decade [5-18]. *Transcription software in this period was based on different types of AI models, including one or more of the following: automated speech recognition, natural language processing, probabilistic graphical models (conditional random field), or logistic regression models [5].

The conceptualization of ambient clinical documentation has evolved in parallel with the technology over the past several years (Figure 1), with a clear bibliometric trend of increasing research on the subject. There is no taxonomy of AI scribe technologies, although one appears to be emerging. One general operating definition of “digital scribe” is the use of automatic speech recognition technology or natural language processing to support clinical documentation [3]. Although the digital scribe concept does not explicitly exclude the use of AI technology, it is only in more recent years that AI has been explicitly labeled as a component of clinical documentation tools. One published literature review subdivided AI-driven documentation systems into generative AI and ambient AI, even if ambient AI may also make use of architecture comparable to that of generative AI [4].

Coiera et al [19] envisioned a progression through the following three stages: (1) human-led systems augmented by tools like dictation and templates (2); mixed-initiative systems where AI assists in converting conversations into summaries; and (3) computer-led systems that autonomously handle documentation, seeking human input only for exceptions. It would seem that in the last 3 years, health care organizations and physicians have been firmly shifting into the second stage, driven by the increasingly advanced technologies available and the salient burdens on clinical and patient care time, particularly in the United States, which has generated the largest proportion of published original research studies on ambient AI scribes. Professional societies and physician groups around the world are also engaged in the global dialogue on the role and promise of AI scribes in medical practice [20-22]. Ultimately, intelligent clinical environments, which capture and integrate data into the EHR, offer the promise of off-loading human-led documentation tasks onto a machine and doing so with an ability to integrate multimodal data from various sources, thereby freeing up the human in the loop and allowing them to focus cognitive efforts on clinical and medical decision-making. So far, initial evaluations have come from small-scale, short-term pilot studies [12,13] that often have volunteer participants who may be biased toward technology.

This excitement has no doubt been spurred by the introduction and widespread availability of large language models (LLMs), which, in terms of development and adoption, have rapidly outpaced other AI technologies underlying documentation support. Numerous industry products [1,23] and a rapidly increasing number of publications have emerged regarding ambient AI scribes using LLMs, even though other AI technologies for documentation tasks have been studied and used for the last 2 decades. A simple PubMed literature search on June 1, 2025, resulted in the retrieval of 940 potentially relevant articles published in the last 10 years (Multimedia Appendix 1); of the most relevant articles (ie, based on their titles), 7 have been published in 2025 (as of the search date) [12,13,17,18,23-25], 8 were published in 2024 [14,15,26-31], and 3 were published in 2023 [10,32,33]. Since this search, further studies have been published to guide ambient digital scribe evaluation [5,34]. During the course of preparing this editorial, authors identified more peer-reviewed literature on ambient AI scribes every few days. Undoubtedly, additional research is forthcoming as hype, hope, and operational needs coincide to drive further adoption.

Despite the rapidly growing published literature on ambient AI scribes and AI-driven documentation, we found that many still focus on similar objectives and evaluation metrics. Consequently, we felt that a call for papers on the topic in *JMIR Medical Informatics* would be valuable for collecting and publishing scientific studies and evidence-based perspectives on broader aspects of ambient AI scribe technologies. As a starting point, we synthesized recent literature about their impact on clinical workflows, well-being, note quality, user experience, patient interaction, and medicolegal aspects, identifying opportunities for further investigation in this field (Multimedia Appendices 2-4).

## Ambient AI Scribe Opportunities

The enthusiasm surrounding AI scribes is tempered by caution. An initial focus of evaluating the outputs of AI scribes is the quality of the note [4], which is assessed by using instruments such as the Physician Documentation Quality Instrument (PDQI-9) [35] or Sheffield Assessment Instrument for Letters (SAIL) [36]; usability is assessed with the NASA Task Load Index (NASA-TLX) [37], and burnout is assessed with various inventories. Results suggest that there is risk for hallucinations or fictitious information [4]. Other evaluations of stand-alone tools for audio recordings suggest potentially high rates of errors, including incorrect information, omissions, and hallucinations [11,29,38]. The high rates of omissions and hallucinations found in some studies underscore the need to evaluate for potential diagnostic errors or other long-term safety risks stemming from AI-generated content [4,10,29-32,39-42]. Others express caution regarding the potential for security risks or risks to medical decision-making in cases where LLMs can access and modify sensitive patient data [31]. Overviews on the topic emphasize the need for careful consideration of the ethical and practical integration of LLMs into clinical practice [1,4], cautioning about potential risks, such as automation bias, privacy concerns, and medicolegal implications [19].

Another major unexamined area is the impact on tangible clinical outcomes and patient safety. Although efficiency, time savings, and productivity are important, there are other measures that are also important. Assessments of note quality, accuracy, and impact on patient safety are essential; Gellert [32] raised a crucial point—there is currently no systematic data collection for evaluating the extent to which clinical errors or negative patient outcomes can be attributed to the use of medical scribes. More comprehensive evaluations of the safe and effective implementation of ambient AI scribe technologies (eg, along the dimensions of the seminal sociotechnological model of health information technology) are still lacking [43]. Additional diversity of clinical specialty applications, clinical disciplines, and practice settings also would be insightful. We identified studies published in a dermatology and urology journal but were unable to retrieve the full-text articles. One pediatrics application indicated positive outcomes during a digital AI scribe pilot in an outpatient pediatric setting [44]. Further, two conference proceeding papers described LLM applications in nursing documentation [27,45], as did a position paper from the Nursing and Artificial Intelligence Leadership Collaborative regarding multimodal LLM support for nursing documentation [46], although these stopped short of discussing AI scribe applications.

The most frequently mentioned gap is the limited study of patients’ or caregivers’ perspectives regarding AI scribes. Most studies rely on clinicians’ perceptions of the patient experience, with very few directly capturing patient viewpoints. Additional research could directly measure patients’ experiences, preferences, and patient-reported outcomes [4,47], especially given recent studies solely examining physicians’ experiences of the patient-physician visit. Pelletier et al [44] incorporated the assessment of caregiver satisfaction in pediatrics, with the sole statistically significant finding being that caregivers’ “provider-specific likelihood to recommend” was higher after the pilot digital scribe implementation. There are also potential patient benefits that remain unexplored. AI scribes may prove useful in providing rapid, patient-friendly visit summaries; outlining the diagnosis and management plan; scheduling appointments; and providing reasons to seek follow-up care [31]. They might also help to bridge communication gaps by highlighting misunderstandings or discrepancies between patient-reported details and those documented in EHRs [45].

The downstream effects of AI-generated notes on clinical communication and reasoning are also unknown. Note bloat—the well-established phenomenon of creating lengthy clinical documentation, which is most often attributed to the copy-paste phenomenon of digitizing documentation [42,48,49]—may also result from AI scribe use; whether large quantities of text redundancy change as a result of AI scribe use is unknown. With regard to clinical reasoning and cognition, as per preliminary evidence from nonmedical studies, such as a study by Kosmyna et al [50], one potential consequence of using generative AI for a writing task is “cognitive debt,” where repeated reliance on AI for cognitive tasks may lead to the atrophy of critical thinking and memory skills. The study found that LLM use impaired memory recall and reduced the brain’s neural engagement with the studied task. Although potentially dull, the documentation process may have beneficial effects on retention and memory. Learner experiences and consequences on expertise development, such as the formative diagnostic process for trainees, are underexplored [51].

Finally, system-level and economic outcomes require more rigorous investigation. Studies call for comprehensive cost-benefit analyses to justify the significant expense of AI scribe technologies [17]. On a broader scale, Gellert [32] cautioned about a largely unstudied systemic risk—the widespread adoption of scribes may decouple physicians from their EHRs and thereby impede the user-driven feedback necessary for the long-term evolution of next-generation clinical AI. In clinical informatics networks, user engagement in the co-design and development of AI scribes is seen as an essential component of the appropriate advancement and adoption of the technology, yet only one study has taken steps to pursue this ideal in evaluating primary care physician needs [9].

## Conclusions

Ambient AI scribes, particularly with the widespread availability of LLMs, offer potential solutions to a previously difficult-to-bridge technological gap in clinical documentation. Although many health systems and physicians are welcoming this potentially practice-changing technology, there are many questions and areas that remain to be fully understood. Substantial clinical documentation also occurs adjacent to or outside of a clinical visit, and the applications of ambient AI scribes and AI-driven documentation technologies in these areas are yet to be explored. Such areas involve various clinician types and health professionals, clinical settings, or community settings. As editors, we look forward to the opportunity to advance the science and understanding of these fields through publishing high-quality and rigorous scholarly work in the *JMIR Medical Informatics* call for papers on “Ambient AI Scribes and AI-Driven Documentation Technologies” [2].

## Supplementary material

10.2196/80898 Multimedia Appendix 1 PubMed search and Gemini 2.5 Pro prompts and responses.

10.2196/80898 Multimedia Appendix 2 NotebookLM prompts and responses.

10.2196/80898 Multimedia Appendix 3 NotebookLM mind map: challenges and limitations.

10.2196/80898 Multimedia Appendix 4 NotebookLM mind map: future directions and recommendations.

## Abbreviations

AI: artificial intelligence
EHR: electronic health record
LLM: large language model
NASA-TLX: NASA Task Load Index
PDQI-9: Physician Documentation Quality Instrument
SAIL: Sheffield Assessment Instrument for Letters
