# Supplementary material for: AI Scribes in Health Care: Balancing Transformative Potential With Responsible Integration
Source: JMIR Med Inform. 2025 Aug 1;13:e80898. doi: 10.2196/80898 (PMC12316405; doi:10.2196/80898)
Supplement: Multimedia Appendix 1 [file medinform-v13-e80898-s001.docx]

## Multimedia Appendix 1. PubMed search and Gemini 2.5 Pro Prompts and Responses

### **Step 1. PubMed search for ambient AI scribe technology**

*Authors’ note: This search was run on June 1, 2025 by author TIL.*

(Charting[Text word] or Notes[Text Word] OR Scribe[Text Word] OR documentation[Text word]) AND (( "Artificial Intelligence"[Mesh] OR "Generative Artificial Intelligence"[Mesh] )) AND ( "Electronic Health Records"[Mesh] OR "Medical Records"[Mesh] OR "Medical Records Systems, Computerized"[Mesh] OR "Medical Records, Problem-Oriented"[Mesh] OR "Medical Records Department, Hospital"[Mesh] OR "Health Records, Personal"[Mesh] )

### **Step 2. Prompt**

*Authors’ note: This prompt was run on June 20, 2025.*

Summarize the key common themes across all of these articles. Where they provide opportunities or gaps for future research, summarize this separately.

1. Duggan MJ, Gervase J, Schoenbaum A, Hanson W, Howell JT 3rd, Sheinberg M, Johnson KB. Clinician Experiences With Ambient Scribe Technology to Assist With Documentation Burden and Efficiency. JAMA Netw Open. 2025 Feb 3;8(2):e2460637. Doi: 10.1001/jamanetworkopen.2024.60637. PubMed [citation] PMID: 39969880, PMCID: PMC11840636
2. Galloway JL, Munroe D, Vohra-Khullar PD, Holland C, Solis MA, Moore MA, Dbouk RH. Impact of an Artificial Intelligence-Based Solution on Clinicians' Clinical Documentation Experience: Initial Findings Using Ambient Listening Technology. J Gen Intern Med. 2024 Oct;39(13):2625-2627. doi: 10.1007/s11606-024-08924-2. Epub 2024 Jul 9. No abstract available. PubMed [citation] PMID: 38980463, PMCID: PMC11436573
3. Kernberg A, Gold JA, Mohan V. Using ChatGPT-4 to Create Structured Medical Notes From Audio Recordings of Physician-Patient Encounters: Comparative Study. J Med Internet Res. 2024 Apr 22;26:e54419. doi: 10.2196/54419. PubMed [citation] PMID: 38648636, PMCID: PMC11074889
4. Kocaballi AB, Ijaz K, Laranjo L, Quiroz JC, Rezazadegan D, Tong HL, Willcock S, Berkovsky S, Coiera E. Envisioning an artificial intelligence documentation assistant for future primary care consultations: A co-design study with general practitioners. J Am Med Inform Assoc. 2020 Nov 1;27(11):1695-1704. Doi: 10.1093/jamia/ocaa131. PubMed [citation] PMID: 32845984, PMCID: PMC7671614
5. Ma SP, Liang AS, Shah SJ, Smith M, Jeong Y, Devon-Sand A, Crowell T, Delahaie C, Hsia C, Lin S, Shanafelt T, Pfeffer MA, Sharp C, Garcia P. Ambient artificial intelligence scribes: utilization and impact on documentation time. J Am Med Inform Assoc. 2025 Feb 1;32(2):381-385. doi: 10.1093/jamia/ocae304. PubMed [citation] PMID: 39688515, PMCID: PMC11756633
6. Misurac J, Knake LA, Blum JM. The Effect of Ambient Artificial Intelligence Notes on Provider Burnout. Appl Clin Inform. 2025 Mar;16(2):252-258. Doi: 10.1055/a-2461-4576. Epub 2024 Nov 5. PubMed [citation] PMID: 39500346, PMCID: PMC11922620
7. Schaye V, DiTullio D, Guzman BV, Vennemeyer S, Shih H, Reinstein I, Weber DE, Goodman A, Wu DTY, Sartori DJ, Santen SA, Gruppen L, Aphinyanaphongs Y, Burk-Rafel J. Large Language Model-Based Assessment of Clinical Reasoning Documentation in the Electronic Health Record Across Two Institutions: Development and Validation Study. J Med Internet Res. 2025 Mar 21;27:e67967. Doi: 10.2196/67967. PubMed [citation] PMID: 40117575, PMCID: PMC11971582
8. Seo J, Choi D, Kim T, Cha WC, Kim M, Yoo H, Oh N, Yi Y, Lee KH, Choi E. Evaluation Framework of Large Language Models in Medical Documentation: Development and Usability Study. J Med Internet Res. 2024 Nov 20;26:e58329. doi: 10.2196/58329. PubMed [citation] PMID: 39566044, PMCID: PMC11618017
9. Shah SJ, Devon-Sand A, Ma SP, Jeong Y, Crowell T, Smith M, Liang AS, Delahaie C, Hsia C, Shanafelt T, Pfeffer MA, Sharp C, Lin S, Garcia P. Ambient artificial intelligence scribes: physician burnout and perspectives on usability and documentation burden. J Am Med Inform Assoc. 2025 Feb 1;32(2):375-380. Doi: 10.1093/jamia/ocae295. PubMed [citation] PMID: 39657021, PMCID: PMC11756571
10. Yim WW, Fu Y, Ben Abacha A, Snider N, Lin T, Yetisgen M. Aci-bench: a Novel Ambient Clinical Intelligence Dataset for Benchmarking Automatic Visit Note Generation. Sci Data. 2023 Sep 6;10(1):586. doi: 10.1038/s41597-023-02487-3. PMID: 37673893; PMCID: PMC10482860.

### **Response (Unaltered output)**

*Authors’ note: Below is a verbatim output from Gemini 2.5 Pro, provided for transparency. Superscripts are generated by Gemini to cite specific parts of articles provided with the prompt as sources of the generated statement. No adjustments were made to the output. Authors subsequently verified and updated claims; those may be found in the “Response (clean version)” section located afterwards.*

Based on a collection of recent studies, several key themes emerge regarding the role, impact, and future of ambient artificial intelligence (AI) scribes in clinical settings. These technologies, which listen to patient-doctor conversations to automatically generate clinical notes, are being actively investigated as a tool to combat documentation burden and clinician burnout.

Key Common Themes

Impact on Clinician Burnout and Well-being

A primary driver for adopting ambient AI is the significant burden of clinical documentation, which is a major contributor to physician burnout^1111111111111111^. Multiple studies show that using ambient AI scribes is associated with a significant reduction in burnout and an improvement in well-being.

- A study by Misurac et al. using the Stanford Professional Fulfillment Index (PFI) found that burnout rates among 35 providers decreased from 69% to 43% after a five-week trial^22^. The study noted a particularly strong improvement in scores for interpersonal disengagement^3333^.
- Shah et al. also found large, statistically significant reductions in both physician task load and burnout as measured by the PFI's work exhaustion sub-scale^444444444^.
- Similarly, a survey by Galloway et al. found that the percentage of clinicians who felt their documentation process had a negative impact on their well-being dropped from 71% to 38.7% after implementation^555^.
- Duggan et al. reported that clinicians felt a lower mental burden from documentation, as it eliminated the difficult task of writing a note from scratch and helped them recall details^666666666^.

Efficiency and Documentation Time

A consistent finding across several studies is that ambient AI scribes can improve efficiency and reduce the time spent on documentation, particularly after hours.

- Duggan et al. found the use of an ambient scribe was associated with 20.4% less time spent in notes per appointment, a 9.3% increase in same-day appointment closure, and 30.0% less after-hours work time^7^.
- Ma et al. quantified a median reduction of 0.57 minutes per note, 6.89 minutes in daily documentation time, and 5.17 minutes in after-hours EHR time^8^.
- Shah et al. reported a median perceived time savings of 20 minutes per half-day of clinic^9^.
- Despite these quantitative gains, feedback can be mixed. Some clinicians noted that time savings were partially offset by the need to substantially edit and proofread the AI-generated notes^101010101010101010^.

Note Quality, Accuracy, and Errors

The quality and accuracy of AI-generated notes is a complex and recurring theme, with findings varying significantly based on whether the study used a commercial tool or evaluated a general large language model (LLM).

- **Perceived Quality of Commercial Tools:** Clinicians in studies using commercial ambient scribes generally report that the quality is acceptable or improved, though editing is required^111111111111111111^. Clinicians in one study rated the AI-generated notes an average of 4.37 out of 5 stars^12^.
- **Objective Accuracy of LLMs:** Studies that tested the underlying technology, such as ChatGPT-4, on simulated transcripts revealed significant problems. Kernberg et al. found an average of 23.6 errors per case, with omissions being the most common (86%), followed by additions or "hallucinations" (10.5%)^13^. The study concluded that the quality and reliability of these notes do not yet meet the standards required for clinical use^14^.
- **Error Frameworks:** To address this, some research has focused on creating frameworks to classify errors. Seo et al. identified seven key error types, finding that "structural malformation" errors had the most significant negative impact on a note's clinical evaluation score^15151515^. This highlights that not just content, but the organization of the note is critical^16^.

User Experience and Heterogeneity of Benefit

The usability of commercial ambient AI tools is generally rated favorably by clinicians^171717171717171717^. However, the technology is not a universal solution, and its benefit can vary significantly among users.

- **Varied Adoption:** Ma et al. observed robust overall utilization (55.25% of encounters) but with significant heterogeneity among individual physicians^18181818^. Some users in the study relinquished their licenses due to low perceived utility^19^. This mixed sentiment was also reflected in a Net Promoter Score (NPS) of 0 in the study by Duggan et al., indicating an equal number of promoters and detractors^20^.
- **"Dose-Response" Effect:** The benefits may correlate with usage. Misurac et al. found a "dose-response" relationship, where clinicians who used the tool more frequently experienced a greater reduction in burnout^21^.
- **Fit for Purpose:** The technology may be a better fit for some specialties than others. Clinicians in fields that rely on highly specific templates (e.g., prenatal care) may find current tools unsatisfying^22^.

Impact on the Patient-Doctor Interaction

A commonly cited benefit of ambient AI is the potential to improve the quality of patient-clinician interactions by freeing the physician from the distraction of a computer screen.

- Clinicians reported being more focused, engaged, and able to have better face-to-face conversations with their patients^232323232323232323^.
- This perception is supported by the finding in Misurac et al. of a robust and significant improvement in "interpersonal disengagement" scores^24242424^.
- Galloway et al. also found that the proportion of clinicians who felt their documentation process negatively impacted the patient experience dropped from 35.5% to just 6.5%^25^.

Medico-Legal Concerns and Professional Autonomy

While AI assistants offer efficiency, they also introduce new concerns regarding professional autonomy and liability.

- In a co-design workshop, general practitioners raised concerns about the medico-legal implications of a constantly recorded, comprehensive consultation record that could be scrutinized in retrospect^26262626^.
- Participants voiced fears of "automation bias," where they might over-rely on the AI's suggestions or hesitate to override them, and coined the term "doctor safety" to refer to the need to protect clinicians in this new environment^27272727^.
- These concerns highlight a tension between AI as an assistant and AI as a potential auditor or supervisor^28^.

Opportunities and Gaps for Future Research

The provided articles consistently identify several critical areas where more research is needed before ambient AI scribes can be responsibly and effectively implemented at scale.

- **Patient Experience and Outcomes:** The most frequently mentioned gap is the lack of data from the patient's perspective. Most studies rely on clinician perceptions of the patient experience. Future research must directly survey patients and measure their experience, preferences, and outcomes to ensure these tools are not negatively impacting care^2929292929292929292929^.
- **Objective Note Quality and Patient Safety:** While clinicians may find the notes adequate, a rigorous and objective assessment of their quality, accuracy, and impact on patient safety is essential^30303030^. The high rate of omissions and hallucinations found in some studies underscores the need to evaluate for potential diagnostic errors or other safety risks stemming from AI-generated content^31313131^.
- **Long-Term, Large-Scale, and Comparative Studies:** Many of the initial findings come from small-scale, short-term pilot studies, often with volunteer participants who may be biased toward technology^323232323232323232^. There is a clear need for longitudinal trials to assess the long-term effects on burnout and efficiency, as well as larger, randomized controlled trials across diverse settings to validate the findings and ensure generalizability^333333333333333333^. Studies comparing the performance of multiple different ambient AI tools are also needed^34^.
- **Cost-Benefit and Return on Investment (ROI) Analysis:** The high cost of ambient AI technology is noted as a significant barrier to widespread adoption^35^. Thorough ROI analyses are required to help healthcare organizations make informed decisions, weighing the financial costs against benefits in efficiency, provider retention, and potentially improved care^36^.
- **Understanding Heterogeneity and Optimization:** Future work should aim to identify which clinicians, specialties, and workflows benefit most from ambient scribes to allow for more strategic and effective implementation^37373737^. Co-design studies involving clinicians in the development process could help create tools that are better adapted to their needs^38383838^.
- **Broader AI Capabilities and Integration:** Research should explore the impact of seamless EHR integration and expanded AI capabilities beyond note generation, such as assisting with order entry, generating patient-friendly summaries, or assessing the quality of clinical reasoning within notes^393939393939393939^. The creation of shared, open datasets like ACI-BENCH is crucial for the scientific community to benchmark and advance these capabilities^40404040^.

### **Response (clean version)**

*Authors’ note: Using the above output as a starting point, authors manually validated and summarized each of Gemini’s claims by reviewing the supporting literature. This was necessary to avoid hallucinations and ensure references had been correctly cited. In some cases, additional manuscripts were found which, although they had not been used in the original prompt, supported the claims or added additional information to the summary. This combined narrative is provided below.*

#### Impact on Clinician Burnout and Well-being

A primary driver for adopting ambient AI is the significant burden of clinical documentation, which is a major contributor to physician burnout [[1–4]](https://paperpile.com/c/2o2RWp/RMBi6+b5wN2+u8qJN+ahzza). Multiple studies indicate that the use of ambient AI scribes is associated with a notable reduction in burnout and an improvement in overall well-being. For instance, Misurac et al. (2025) found that burnout rates among 35 providers decreased from 69% to 43% after a five-week trial, with a particularly strong improvement observed in scores for interpersonal disengagement [[3]](https://paperpile.com/c/2o2RWp/u8qJN). Similarly, Shah et al. (2025) reported large, statistically significant reductions in both physician task load and burnout as measured by the Professional Fulfillment Index's (PFI) work exhaustion sub-scale [[2]](https://paperpile.com/c/2o2RWp/b5wN2). Furthermore, a survey by Galloway et al. (2024) revealed that the percentage of clinicians who felt their documentation process negatively impacted their well-being dropped significantly from 71% to 38.7% after implementation [[5]](https://paperpile.com/c/2o2RWp/tXIVe). Duggan et al. (2025) also noted that clinicians experienced a lower mental burden from documentation, as the technology eliminated the difficult task of writing notes from scratch and aided in recalling details [[4]](https://paperpile.com/c/2o2RWp/ahzza).

#### Efficiency and Documentation Time

A consistent finding across several studies is that ambient AI scribes can enhance efficiency and reduce the time spent on documentation, especially after regular work hours. Duggan et al. observed that the use of an ambient scribe was associated with a 20.4% reduction in time spent on notes per appointment, a 9.3% increase in same-day appointment closure, and a 30.0% decrease in after-hours work time [[4]](https://paperpile.com/c/2o2RWp/ahzza). Ma et al. (2025) also quantified these types of gains, reporting a median reduction of 0.57 minutes per note, 6.89 minutes in daily documentation time, and 5.17 minutes in after-hours electronic health record (EHR) time [[1]](https://paperpile.com/c/2o2RWp/RMBi6). Additionally, Shah et al (2025). reported a median perceived time savings of 20 minutes per half-day of clinic [[2]](https://paperpile.com/c/2o2RWp/b5wN2). Similarly, a study at Chi Mei Medical Center in Taiwan addressed the "demanding nature of nursing documentation" by implementing a ChatGPT-based tool called "A+ Nurse". The initiative was aimed at reducing overall nursing workloads and the tool successfully reduced the time nurses spent on documentation from 15 minutes down to approximately 5 minutes per patient without an apparent decrease in quality [[6]](https://paperpile.com/c/2o2RWp/9Ta4l).

Despite these quantitative improvements, feedback can be mixed, with some clinicians noting that the time saved was partially offset by the need to extensively edit and proofread the AI-generated notes [[4,7]](https://paperpile.com/c/2o2RWp/ahzza+cE3MM).

#### Note Quality, Accuracy, and Errors

The quality and accuracy of AI-generated notes present a recurring concern. Most studies report the editing of AI-generated notes by clinicians is necessary [[3,4,7–9]](https://paperpile.com/c/2o2RWp/ahzza+cE3MM+u8qJN+LPqJZ+9Q4CG). However, the quality of notes varied significantly depending on whether a commercial tool or a general large language model (LLM) was evaluated. Misurac et al. (2025) reported that users rated commercial tools quite favourably [[3]](https://paperpile.com/c/2o2RWp/u8qJN). In an evaluation study of a commercial product in a controlled setting [[10,11]](https://paperpile.com/c/2o2RWp/3zA7A+mh9b0), the rates of clinically non-significant and clinically significant hallucinations were 0.5% and 0.21%, respectively, Conversely, when using ChatGPT-4 to create structured medical notes from audio recordings of physician-patient encounters, Kernberg et al. (2024) found an average 23.6 errors per case, with omissions being the most common (86%), followed by addition errors (10.5%), and inclusion of incorrect facts (3.2%) [[12]](https://paperpile.com/c/2o2RWp/78DkG). That study concluded that the quality and reliability of these notes do not yet meet the standards required for clinical use [[12]](https://paperpile.com/c/2o2RWp/78DkG). Performance of ambient AI scribes thus seems to vary widely depending on the scribe and scenario.

To address these issues, research has focused on developing frameworks to classify errors. Seo et al. (2024) identified seven key error types, determining that "structural malformation" errors had the most significant negative impact on a note's clinical evaluation score, underscoring the critical importance of note organization beyond just content [[13]](https://paperpile.com/c/2o2RWp/3BiQI).

#### User Experience and Heterogeneity of Benefit

The usability of commercial ambient AI tools is generally rated favorably by clinicians. However, the technology is not a universal solution, and its benefits can vary significantly among users. Shah et al. (2025) found that clinicians generally viewed the ambient AI scribe tool's impact on patient engagement positively, with 38 of 56 interview comments (68%) reflecting this sentiment. However, adoption of these tools faced barriers such as limited functionality for non-English speaking patients and accessibility issues for physicians lacking a compatible device. Despite some positive impressions, physician perspectives on accuracy and style were largely negative, particularly regarding note length and editing requirements [[14]](https://paperpile.com/c/2o2RWp/bdHlU), highlighting the mixed impressions created by these tools. Ma et al. (2025) observed robust overall utilization (55.25% of encounters) but with considerable heterogeneity among individual physicians [[1]](https://paperpile.com/c/2o2RWp/RMBi6). Some users in their study even relinquished their licenses due to low perceived utility. This mixed sentiment was also reflected in a Net Promoter Score (NPS) of 0 in the study by Duggan et al. (2025), indicating an equal number of promoters and detractors [[4]](https://paperpile.com/c/2o2RWp/ahzza). As with all tools, familiarity and usage may play a role; Misurac et al. (2025) found a "dose-response" relationship where clinicians who used the tool more frequently experienced a greater reduction in burnout [[3]](https://paperpile.com/c/2o2RWp/u8qJN). Furthermore, the technology may be a better fit for some specialties than others, with clinicians in fields reliant on highly specific templates (e.g., prenatal care) potentially finding current tools unsatisfying [[4]](https://paperpile.com/c/2o2RWp/ahzza). Future work may wish to identify which clinicians, specialties, and workflows benefit most from ambient scribes to allow for more strategic and effective implementation

#### Impact on the Patient-Doctor Interaction

A commonly cited benefit of ambient AI is its potential to improve the quality of patient-clinician interactions by freeing the physician from the distraction of a computer screen. Clinicians have reported feeling more focused, engaged, and able to have better face-to-face conversations with their patients [[3,4,14,15]](https://paperpile.com/c/2o2RWp/IGUhc+ahzza+u8qJN+bdHlU). This perception is supported by Misurac et al.'s (2025) finding of a robust and significant improvement in "interpersonal disengagement" scores [[3]](https://paperpile.com/c/2o2RWp/u8qJN). Additionally, Galloway et al. found that the proportion of clinicians who felt their documentation process negatively impacted the patient experience dropped dramatically from 35.5% to just 6.5% [[5]](https://paperpile.com/c/2o2RWp/tXIVe).

#### Medico-Legal Concerns and Professional Autonomy

While AI assistants offer efficiency, they also introduce new concerns regarding professional autonomy and liability. Participants voiced fears of "automation bias," where they might over-rely on AI suggestions, and coined the term "doctor safety" to refer to the need to protect clinicians in this new environment. This highlights a tension between AI as an "assistant" versus an "auditor". A separate study using EEG to measure brain activity during an essay-writing task warned of a potential "cognitive debt," where reliance on an LLM assistant systematically reduced cognitive engagement and weakened neural connectivity. This cognitive offloading was linked to impaired memory recall and a diminished sense of ownership over the final work product, raising alarms about the long-term impact of over-reliance on AI in a field that demands constant critical thinking [[16]](https://paperpile.com/c/2o2RWp/2LzKg).

### **References**

1. [Ma SP, Liang AS, Shah SJ, Smith M, Jeong Y, Devon-Sand A, Crowell T, Delahaie C, Hsia C, Lin S, Shanafelt T, Pfeffer MA, Sharp C, Garcia P. Ambient artificial intelligence scribes: utilization and impact on documentation time. J Am Med Inform Assoc 2025 Feb 1;32(2):381–385. PMID:39688515](http://paperpile.com/b/2o2RWp/RMBi6)

2. [Shah SJ, Devon-Sand A, Ma SP, Jeong Y, Crowell T, Smith M, Liang AS, Delahaie C, Hsia C, Shanafelt T, Pfeffer MA, Sharp C, Lin S, Garcia P. Ambient artificial intelligence scribes: physician burnout and perspectives on usability and documentation burden. J Am Med Inform Assoc 2025 Feb 1;32(2):375–380. PMID:39657021](http://paperpile.com/b/2o2RWp/b5wN2)

3. [Misurac J, Knake LA, Blum JM. The Effect of Ambient Artificial Intelligence Notes on Provider Burnout. Appl Clin Inform 2025 Mar;16(2):252–258. PMID:39500346](http://paperpile.com/b/2o2RWp/u8qJN)

4. [Duggan MJ, Gervase J, Schoenbaum A, Hanson W, Howell JT 3rd, Sheinberg M, Johnson KB. Clinician experiences with ambient scribe technology to assist with documentation burden and efficiency. JAMA Netw Open American Medical Association (AMA); 2025 Feb 3;8(2):e2460637. PMID:39969880](http://paperpile.com/b/2o2RWp/ahzza)

5. [Galloway JL, Munroe D, Vohra-Khullar PD, Holland C, Solis MA, Moore MA, Dbouk RH. Impact of an Artificial Intelligence-Based Solution on Clinicians’ Clinical Documentation Experience: Initial Findings Using Ambient Listening Technology. J Gen Intern Med 2024 Oct;39(13):2625–2627. PMID:38980463](http://paperpile.com/b/2o2RWp/tXIVe)

6. [Chen C-J, Liao C-T, Tung Y-C, Liu C-F. Enhancing Healthcare Efficiency: Integrating ChatGPT in Nursing Documentation. Stud Health Technol Inform 2024 Aug 22;316:851–852. PMID:39176926](http://paperpile.com/b/2o2RWp/9Ta4l)

7. [Blaseg E, Huffstetler A. Artificial Intelligence Scribes Shape Health Care Delivery. Am Fam Physician 2025 Apr;111(4):304–305. PMID:40238969](http://paperpile.com/b/2o2RWp/cE3MM)

8. [Haberle T, Cleveland C, Snow GL, Barber C, Stookey N, Thornock C, Younger L, Mullahkhel B, Ize-Ludlow D. The impact of nuance DAX ambient listening AI documentation: a cohort study. J Am Med Inform Assoc Oxford University Press (OUP); 2024 Apr 3;31(4):975–979. PMID:38345343](http://paperpile.com/b/2o2RWp/LPqJZ)

9. [Tierney AA, Gayre G, Hoberman B, Mattern B, Ballesca M, Kipnis P, Liu V, Lee K. Ambient artificial intelligence scribes to alleviate the burden of clinical documentation. NEJM Catal Innov Care Deliv Massachusetts Medical Society; 2024 Feb 21;5(3). doi:](http://paperpile.com/b/2o2RWp/9Q4CG) [10.1056/cat.23.0404](http://dx.doi.org/10.1056/cat.23.0404)

10. [Balloch J, Sridharan S, Oldham G, Wray J, Gough P, Robinson R, Sebire NJ, Khalil S, Asgari E, Tan C, Taylor A, Pimenta D. Use of an ambient artificial intelligence tool to improve quality of clinical documentation. Future Healthc J Elsevier BV; 2024 Sep 1;11(3):100157. PMID:39371531](http://paperpile.com/b/2o2RWp/3zA7A)

11. [Medical AI Healthcare Assistant. Tortus. 2024. Available from:](http://paperpile.com/b/2o2RWp/mh9b0) <https://tortus.ai/> [[accessed Jul 7, 2025]](http://paperpile.com/b/2o2RWp/mh9b0)

12. [Kernberg A, Gold JA, Mohan V. Using ChatGPT-4 to create structured medical notes from audio recordings of physician-patient encounters: Comparative study. J Med Internet Res JMIR Publications Inc.; 2024 Apr 22;26:e54419. PMID:38648636](http://paperpile.com/b/2o2RWp/78DkG)

13. [Seo J, Choi D, Kim T, Cha WC, Kim M, Yoo H, Oh N, Yi Y, Lee KH, Choi E. Evaluation framework of large language models in medical documentation: Development and usability study. J Med Internet Res JMIR Publications Inc.; 2024 Nov 20;26(1):e58329. PMID:39566044](http://paperpile.com/b/2o2RWp/3BiQI)

14. [Shah SJ, Crowell T, Jeong Y, Devon-Sand A, Smith M, Yang B, Ma SP, Liang AS, Delahaie C, Hsia C, Shanafelt T, Pfeffer MA, Sharp C, Lin S, Garcia P. Physician Perspectives on Ambient AI Scribes. JAMA Netw Open 2025 Mar 3;8(3):e251904. PMID:40126477](http://paperpile.com/b/2o2RWp/bdHlU)

15. [Bundy H, Gerhart J, Baek S, Connor CD, Isreal M, Dharod A, Stephens C, Liu T-L, Hetherington T, Cleveland J. Can the administrative loads of physicians be alleviated by AI-facilitated clinical documentation? J Gen Intern Med Springer Science and Business Media LLC; 2024 Nov 27;39(15):2995–3000. PMID:38937369](http://paperpile.com/b/2o2RWp/IGUhc)

16. [Kocaballi AB, Ijaz K, Laranjo L, Quiroz JC, Rezazadegan D, Tong HL, Willcock S, Berkovsky S, Coiera E. Envisioning an artificial intelligence documentation assistant for future primary care consultations: A co-design study with general practitioners. J Am Med Inform Assoc 2020 Nov 1;27(11):1695–1704. PMID:32845984](http://paperpile.com/b/2o2RWp/2LzKg)
