# Supplementary material for: AI Scribes in Health Care: Balancing Transformative Potential With Responsible Integration
Source: JMIR Med Inform. 2025 Aug 1;13:e80898. doi: 10.2196/80898 (PMC12316405; doi:10.2196/80898)
Supplement: Multimedia Appendix 2 [file medinform-v13-e80898-s002.docx]

## Multimedia Appendix 2. NotebookLM Prompts and Responses

### **Initial Prompt**

*Author’s note: 28 articles * were provided to NotebookLM to complete the prompted task. Some articles could not be obtained (not open access or otherwise retrievable by the authors). The prompts below were run on July 12, 2025.*

For all of the provided articles, summarize the key common themes from these articles. Where they provide opportunities or gaps for future research, summarize this separately.

* 28 references provided:

1. Balloch J, Sridharan S, Oldham G, Wray J, Gough P, Robinson R, Sebire NJ, Khalil S, Asgari E, Tan C, Taylor A, Pimenta D. Use of an ambient artificial intelligence tool to improve quality of clinical documentation. Future Healthc J.2024 Jun 26;11(3):100157. doi: 10.1016/j.fhj.2024.100157. eCollection 2024 Sep. Erratum in: Future Healthc J. 2025 Apr 30;12(2):100247. doi:10.1016/j.fhj.2025.100247.. PubMed [citation] PMID: 39371531, PMCID: PMC11452835
2. Blaseg E, Huffstetler A. Artificial Intelligence Scribes Shape Health Care Delivery. Am Fam Physician. 2025 Apr;111(4):304-305. PubMed [citation] PMID:40238969
3. Bracken A, Reilly C, Feeley A, Sheehan E, Merghani K, Feeley I. Artificial Intelligence (AI) - Powered Documentation Systems in Healthcare: A Systematic Review. J Med Syst. 2025 Feb 18;49(1):28. doi: 10.1007/s10916-025-02157-4. Review. PubMed [citation] PMID: 39966286, PMCID: PMC11835907
4. Bundy H, Gerhart J, Baek S, Connor CD, Isreal M, Dharod A, Stephens C, Liu TL, Hetherington T, Cleveland J. Can the Administrative Loads of Physicians be Alleviated by AI-Facilitated Clinical Documentation? J Gen Intern Med. 2024 Nov;39(15):2995-3000. doi: 10.1007/s11606-024-08870-z. Epub 2024 Jun 27. PubMed [citation] PMID: 38937369, PMCID: PMC11576703
5. Chen CJ, Liao CT, Tung YC, Liu CF. Enhancing Healthcare Efficiency: Integrating ChatGPT in Nursing Documentation. Stud Health Technol Inform. 2024 Aug 22;316:851-852. doi: 10.3233/SHTI240545. PubMed [citation] PMID: 39176926
6. Duggan MJ, Gervase J, Schoenbaum A, Hanson W, Howell JT 3rd, Sheinberg M, Johnson KB. Clinician Experiences With Ambient Scribe Technology to Assist With Documentation Burden and Efficiency. JAMA Netw Open. 2025 Feb 3;8(2):e2460637. Doi: 10.1001/jamanetworkopen.2024.60637. PubMed [citation] PMID: 39969880, PMCID: PMC11840636
7. Galloway JL, Munroe D, Vohra-Khullar PD, Holland C, Solis MA, Moore MA, Dbouk RH. Impact of an Artificial Intelligence-Based Solution on Clinicians' Clinical Documentation Experience: Initial Findings Using Ambient Listening Technology. J Gen Intern Med. 2024 Oct;39(13):2625-2627. doi: 10.1007/s11606-024-08924-2. Epub 2024 Jul 9. No abstract available. PubMed [citation] PMID: 38980463, PMCID: PMC11436573
8. Gellert GA. Medical Scribes: Symptom or Cause of Impeded Evolution of a Transformative Artificial Intelligence in the Electronic Health Record? Perspect Health Inf Manag. 2023 Jan 10;20(1):1d. eCollection 2023 Winter. PubMed [citation] PMID: 37215336, PMCID: PMC9860472
9. Gupta P, Zhang Z, Song M, Michalowski M, Hu X, Stiglic G, Topaz M. Rapid Review: Growing Usage of Multimodal Large Language Models in Healthcare. 2025.
10. Haberle T, Cleveland C, Snow GL, Barber C, Stookey N, Thornock C, Younger L, Mullahkhel B, Ize-Ludlow D. The impact of nuance DAX ambient listening AI documentation: a cohort study. J Am Med Inform Assoc. 2024 Apr 3;31(4):975-979. doi: 10.1093/jamia/ocae022. PubMed [citation] PMID: 38345343, PMCID: PMC10990544
11. Kernberg A, Gold JA, Mohan V. Using ChatGPT-4 to Create Structured Medical Notes From Audio Recordings of Physician-Patient Encounters: Comparative Study. J Med Internet Res. 2024 Apr 22;26:e54419. doi: 10.2196/54419. PubMed [citation] PMID: 38648636, PMCID: PMC11074889
12. Kocaballi AB, Ijaz K, Laranjo L, Quiroz JC, Rezazadegan D, Tong HL, Willcock S, Berkovsky S, Coiera E. Envisioning an artificial intelligence documentation assistant for future primary care consultations: A co-design study with general practitioners. J Am Med Inform Assoc. 2020 Nov 1;27(11):1695-1704. Doi: 10.1093/jamia/ocaa131. PubMed [citation] PMID: 32845984, PMCID: PMC7671614
13. Ma SP, Liang AS, Shah SJ, Smith M, Jeong Y, Devon-Sand A, Crowell T, Delahaie C, Hsia C, Lin S, Shanafelt T, Pfeffer MA, Sharp C, Garcia P. Ambient artificial intelligence scribes: utilization and impact on documentation time. J Am Med Inform Assoc. 2025 Feb 1;32(2):381-385. doi: 10.1093/jamia/ocae304. PubMed [citation] PMID: 39688515, PMCID: PMC11756633
14. Michalowski M, Topaz M, Peltonen LM. An AI-Enabled Nursing Future With no Documentation Burden: A Vision for a New Reality. J Adv Nurs. 2025 Mar 24. Doi: 10.1111/jan.16911. [Epub ahead of print] PubMed [citation] PMID: 40129115
15. Misurac J, Knake LA, Blum JM. The Effect of Ambient Artificial Intelligence Notes on Provider Burnout. Appl Clin Inform. 2025 Mar;16(2):252-258. Doi: 10.1055/a-2461-4576. Epub 2024 Nov 5. PubMed [citation] PMID: 39500346, PMCID: PMC11922620
16. Ng JJW, Wang E, Zhou X, Zhou KX, Goh CXL, Sim GZN, Tan HK, Goh SSN, Ng QX. Evaluating the performance of artificial intelligence-based speech recognition for clinical documentation: a systematic review. BMC Med Inform Decis Mak. 2025 Jul 1;25(1):236. doi: 10.1186/s12911-025-03061-0. PubMed [citation] PMID: 40598136, PMCID: PMC12220090
17. Owens LM, Wilda JJ, Grifka R, Westendorp J, Fletcher JJ. Effect of Ambient Voice Technology, Natural Language Processing, and Artificial Intelligence on the Patient-Physician Relationship. Appl Clin Inform. 2024 Aug;15(4):660-667. Doi: 10.1055/a-2337-4739. Epub 2024 Jun 4. PubMed [citation] PMID: 38834180, PMCID: PMC11305826
18. Pelletier JH, Watson K, Michel J, McGregor R, Rush SZ. Effect of a generative artificial intelligence digital scribe on pediatric provider documentation time, cognitive burden, and burnout. JAMIA Open. 2025 Jul 3;8(4):ooaf068. Doi: 10.1093/jamiaopen/ooaf068. eCollection 2025 Aug. PubMed [citation] PMID: 40620477, PMCID: PMC12225174
19. Quiroz JC, Laranjo L, Kocaballi AB, Berkovsky S, Rezazadegan D, Coiera E. Challenges of developing a digital scribe to reduce clinical documentation burden. NPJ Digit Med. 2019 Nov 22;2:114. doi: 10.1038/s41746-019-0190-1. eCollection 2019. Review. PubMed [citation] PMID: 31799422, PMCID: PMC6874666
20. Sasseville M, Yousefi F, Ouellet S, Naye F, Stefan T, Carnovale V, Bergeron F, Ling L, Gheorghiu B, Hagens S, Gareau-Lajoie S, LeBlanc A. The Impact of AI Scribes on Streamlining Clinical Documentation: A Systematic Review. Healthcare (Basel). 2025 Jun 16;13(12):1447. doi: 10.3390/healthcare13121447. PMID: 40565474; PMCID: PMC12193156.
21. Schaye V, DiTullio D, Guzman BV, Vennemeyer S, Shih H, Reinstein I, Weber DE, Goodman A, Wu DTY, Sartori DJ, Santen SA, Gruppen L, Aphinyanaphongs Y, Burk-Rafel J. Large Language Model-Based Assessment of Clinical Reasoning Documentation in the Electronic Health Record Across Two Institutions: Development and Validation Study. J Med Internet Res. 2025 Mar 21;27:e67967. Doi: 10.2196/67967. PubMed [citation] PMID: 40117575, PMCID: PMC11971582
22. Seo J, Choi D, Kim T, Cha WC, Kim M, Yoo H, Oh N, Yi Y, Lee KH, Choi E. Evaluation Framework of Large Language Models in Medical Documentation: Development and Usability Study. J Med Internet Res. 2024 Nov 20;26:e58329. doi: 10.2196/58329. PubMed [citation] PMID: 39566044, PMCID: PMC11618017
23. Shah SJ, Crowell T, Jeong Y, Devon-Sand A, Smith M, Yang B, Ma SP, Liang AS, Delahaie C, Hsia C, Shanafelt T, Pfeffer MA, Sharp C, Lin S, Garcia P. Physician Perspectives on Ambient AI Scribes. JAMA Netw Open. 2025 Mar 3;8(3):e251904. Doi: 10.1001/jamanetworkopen.2025.1904. PubMed [citation] PMID: 40126477, PMCID: PMC11933996
24. Shah SJ, Devon-Sand A, Ma SP, Jeong Y, Crowell T, Smith M, Liang AS, Delahaie C, Hsia C, Shanafelt T, Pfeffer MA, Sharp C, Lin S, Garcia P. Ambient artificial intelligence scribes: physician burnout and perspectives on usability and documentation burden. J Am Med Inform Assoc. 2025 Feb 1;32(2):375-380. Doi: 10.1093/jamia/ocae295. PubMed [citation] PMID: 39657021, PMCID: PMC11756571
25. Stults CD, Deng S, Martinez MC, Wilcox J, Szwerinski N, Chen KH, Driscoll S, Washburn J, Jones VG. Evaluation of an Ambient Artificial Intelligence Documentation Platform for Clinicians. JAMA Netw Open. 2025 May 1;8(5):e258614. doi: 10.1001/jamanetworkopen.2025.8614. PubMed [citation] PMID: 40314951, PMCID: PMC12048851
26. Thawinwisan N, Liu C, Kishimoto K, Yamamoto G, Mori Y, Kuroda T. Comparing Patient Perception and Physician's Records: Generative AI Performance Evaluation. Stud Health Technol Inform. 2024 Aug 22;316:671-675. doi: 10.3233/SHTI240503. PubMed [citation] PMID: 39176831
27. Tierney AA, Gayre G, Hoberman B, Mattern B, BAllesca M, Kipnis P, Liu V, Lee Kristine. Ambient Artificial Intelligence Scribes to Alleviate the Burden of Clinical Documentation. NEJM Catal Innov Care Deliv 2024;5(3). Doi: 10.1056/CAT.23.0404
28. Tripathi S, Sukumaran R, Cook TS. Efficient healthcare with large language models: optimizing clinical workflow and enhancing patient care. J Am Med Inform Assoc. 2024 May 20;31(6):1436-1440. doi: 10.1093/jamia/ocad258. PubMed [citation] PMID: 38273739, PMCID: PMC11105142

### **Initial Response**

The provided articles extensively discuss the application of Artificial Intelligence (AI) in healthcare, particularly focusing on **AI-powered documentation systems**, including ambient AI scribes and various large language models (LLMs) and multimodal large language models (MLLMs).

#### Key Common Themes

1. **Alleviating Documentation Burden and Clinician Burnout**: A primary and consistent theme is that AI-driven documentation systems are being developed and implemented to **reduce the significant administrative burden** associated with clinical documentation, which is a major contributor to healthcare professional (HCP) burnout and dissatisfaction. The goal is to allow clinicians to dedicate more time to direct patient care.
   - AI scribes, utilizing automatic speech recognition (ASR) and natural language processing (NLP), aim to automate and enhance documentation efficiency and accuracy.
   - These tools are hypothesized to improve clinician well-being and job satisfaction.
2. **Efficiency and Time Savings**: Studies frequently report that AI documentation tools, such as ambient AI scribes, are associated with **reduced documentation time** and **decreased after-hours work** .
   - For instance, one study found a 20.4% reduction in time spent on notes per appointment and a 30% reduction in after-hours work time. Another reported a median daily documentation time decrease of 6.89 minutes and after-hours EHR time decrease of 5.17 minutes.
   - Consultation lengths can also be shortened, sometimes by as much as 26.3%, without affecting patient interaction time.
   - The most notable gains in efficiency are seen in the documentation of complex cases.
   - However, results on workflow efficiency are mixed, with some studies finding that post-editing can negate potential time gains. Some clinicians even reported that editing AI-generated content took longer than writing notes from scratch.
3. **Documentation Quality and Accuracy Concerns**: While AI tools show promise in improving and standardizing documentation quality, there are **significant and persistent concerns regarding accuracy, consistency, and the potential for "hallucinations"** (false or fabricated information).
   - Errors can include omissions, incorrect facts, and additions not present in the original conversation. Omission errors are particularly common and pose safety risks.
   - The problem of "note bloat" (inclusion of unnecessary or repetitive details) has also been observed.
   - Accuracy can be as low as 53% in some cases, with substantial variation across replicates. Some AI-generated notes are rated below acceptable quality standards for clinical use.
   - The quality and consistency of AI-generated documentation can vary significantly depending on the specific technology, model training data, and implementation approach. Tools struggle with specialized terminology or accented speech.
   - Hallucination rates have been documented to vary between 3% and 28% in the literature.
4. **Impact on Clinician Experience and Satisfaction**: Many clinicians report **positive experiences** with AI tools, citing ease of use, simple user interfaces, and the aid to note-taking.
   - Ambient AI has been associated with significant reductions in **physician task load** and **cognitive burden**, allowing for a greater sense of engagement with patients.
   - However, some express initial **trust issues** with the technology or find AI-generated notes to be too verbose, not tailored to their specific needs, or require significant editing.
   - While satisfaction has improved, some studies found a **limited impact on reducing burnout**.
5. **Patient Engagement and Experience**: AI scribes can enable physicians to be **more present and engaged during appointments**, fostering more personable provider-patient encounters.
   - Early patient feedback has been generally positive, with some noting improved interaction with their physicians.
   - However, there are instances where patients felt uncomfortable with the technology's use after reviewing the notes. Patient comfort and privacy are crucial considerations.
6. **Rise of Large Language Models (LLMs) and Multimodal LLMs (MLLMs)**: The advancement of powerful LLMs (e.g., GPT-4) is central to AI-facilitated clinical documentation.
   - MLLMs, which integrate multiple data modalities like images, audio, and quantitative data beyond text, are emerging as a transformative technology in healthcare.
   - They show promise in diverse applications such as **medical diagnosis and clinical report generation** (especially in radiology, pathology, ophthalmology), surgical assistance, public mental health assessment, and secure healthcare data management.
   - MLLM research has surged, particularly from Asia and North America, focusing on integrating diverse data types for comprehensive patient assessment.

#### Opportunities and Gaps for Future Research

The sources highlight several critical areas for further investigation and development:

1. **Improving AI Technology and Performance**:
   - **Refinement of Accuracy and Consistency**: Continued research is needed to refine AI tools to consistently meet high standards in clinical documentation, particularly addressing variability in quality and accuracy and minimizing "hallucinations".
   - **Reproducibility of LLM Outputs**: Studies should investigate whether repeated applications of the same prompts to LLMs yield consistent results, as some data elements are inconsistently reported across replicates.
   - **Handling Complex Data**: Further research is needed to improve AI's effectiveness in handling longer and denser information, such as complex medical cases.
   - **Advanced Capabilities**: Development of AI that can capture non-verbal cues and contextual details from audio/video, integrate medical knowledge, and resolve conflicting information. AI should adapt to various speech styles, accents, and clinical terminologies.
   - **Customization**: Developing tools that can be customized to individual clinician or departmental needs, documentation styles, and specific specialties (e.g., physical exam details).
   - **Real-time Error Correction**: Enhancements for real-time error correction in AI transcription are crucial.
   - **Next-Generation Scribes**: Research into end-to-end AI scribe capabilities that integrate LLM-driven summarization and repurposing of text, rather than just transcription, is recommended.
2. **Broader and Longitudinal Study Design**:
   - **Diverse Populations and Settings**: Future research should encompass consultations across varying patient populations, providing a range of accents, clinical scenarios, and diverse clinical sites across multicultural regions.
   - **Long-term Impacts**: Longitudinal studies are necessary to assess the sustained impact of ambient AI on professional fulfillment and burnout over extended periods.
   - **Widespread Implementation**: Investigate outcomes after large-scale expansion of the technology across entire organizations and in different clinical settings (e.g., emergency departments, inpatient settings).
3. **Impact on Human Roles and Workflows**:
   - **Understanding User Heterogeneity**: Identify which clinicians benefit most from current AI scribe technology and how future iterations can support a broader audience.
   - **Clinician-AI Interaction**: Understanding how physicians review and edit generated text, and the cognitive processes involved in this interaction.
   - **Workflow Integration**: Develop interoperable systems capable of seamlessly integrating with existing EHRs and clinician interfaces.
   - **Impact Beyond Documentation Time**: Explore the relationship between ambient AI usage and other factors such as patient outcomes and clinical efficiency, medical synthesis, clinical communication, and clinical reasoning.
   - **Human-AI Collaboration Models**: Different models of doctor-AI collaboration need to be designed and investigated to ensure patient safety, quality of care, doctor safety, and doctor autonomy. Research into the potential for job displacement and the evolving dynamics of the healthcare workforce in the context of AI adoption is also needed.
4. **Ethical, Regulatory, and Data Considerations**:
   - **Data Availability and Privacy**: Collective efforts are needed to make rich, accurate clinical data available for AI research, while rigorously protecting patient privacy with ethical considerations and HIPAA compliance.
   - **Standardized Evaluation Frameworks**: There is a critical need for harmonized evaluation metrics and structured error analysis protocols for MLLMs to ensure robustness and clinical meaningfulness. This includes developing methods to formally evaluate model explainability through clinician feedback.
   - **Regulatory and Ethical Readiness**: Future studies must address data governance, regulatory compliance (e.g., HIPAA, FDA), fairness audits, and systematic safety and risk mitigation strategies, including handling incidental findings.
   - **Patient Perspectives**: More qualitative research is needed to explore patient perspectives and preferences regarding AI scribe tools to ensure inclusive and equitable development and implementation.
5. **Economic and Organizational Impact**:
   - **Cost-Effectiveness and ROI**: Thorough cost-benefit and return on investment (ROI) analyses are needed to justify the high costs of these technologies for widespread adoption.
   - **Implementation Strategies**: Research on the integration process of these technologies and potential barriers to their adoption in different healthcare environments is important.
   - **Systemic Implications**: Focus on how ambient AI's potential extends beyond individual benefits to broader systemic implications, such as reduced costs associated with staff turnover and absenteeism.
   - **Medical Education**: Further research on the use of LLMs in the EHR for assessment and feedback in medical education can be transformative.

###

### **Follow-up Prompt 1**

Support the claims in this summary more clearly with data and findings from the articles provided.

### **Follow-up Response 1**

The sources collectively illustrate the burgeoning role of AI, particularly ambient AI scribes and Large Language Models (LLMs), in transforming healthcare documentation. They detail the perceived benefits, existing limitations, and the vast potential for future advancements.

Here's a comprehensive look at the key common themes, supported by specific findings from the provided articles:

#### Key Common Themes

- **Reduction of Documentation Burden and Physician Burnout**
  - A primary driver for AI adoption is the **overwhelming administrative load** associated with clinical documentation, which significantly contributes to healthcare professional (HCP) burnout. HCPs often spend **two hours on average outside their official working day** on documentation tasks.
  - Studies demonstrate that AI tools can **alleviate this burden and reduce cognitive load**. For instance, a pilot study using DAX Copilot found it **reduced documentation time** and **alleviated anxieties of having to retain important clinical details**, contributing to physicians feeling "a little bit less burned out".
  - **Longitudinal studies** observed a statistically significant decrease in physician task load and burnout scores. One study reported a **decrease in burnout from 54.9% to 33.3%** among pediatric providers after digital scribe use. Another indicated that high DAX™ use was associated with **less provider burnout** on the Oldenburg Burnout Inventory disengagement score, with users "less likely to talk about work in a negative way" and "more likely to have enough energy for leisure activities after work".
  - The **cognitive burden of note-writing** significantly decreased, with providers reporting an average **6.9-point reduction** (on a 0-20 scale) in mental demand, an **8.0-point reduction** in feeling rushed, and a **7.0-point reduction** in effort required.
- **Improved Efficiency and Time Savings**
  - AI tools consistently show the potential for **significant time savings** in documentation.
  - One study found that consultations using an ambient AI tool were **significantly shorter overall (delta -193 seconds)** compared to standard EHR use, equating to a **26.3% time-saving**. This efficiency did not significantly reduce patient-facing time.
  - The use of ambient scribing technology was associated with a **20.4% reduction in time spent on notes per appointment** (from 10.3 to 8.2 minutes), **9.3% greater same-day appointment closure** (from 66.2% to 72.4%), and **30.0% less after-hours work time per workday** (from 50.6 to 35.4 minutes).
  - An early pilot demonstrated that ambient AI scribes were widely used (in **55.25% of encounters**) and associated with reduced median time per note by **0.57 minutes**, daily documentation time by **6.89 minutes**, and daily total EHR time by **19.95 minutes**.
- **Variable Documentation Quality and Accuracy**
  - While AI tools can standardize documentation quality and potentially reduce misinterpretation, **concerns about accuracy** are prevalent.
  - One study using ChatGPT-4 to generate SOAP notes revealed, on average, **23.6 errors per clinical case**, with **omissions being the most common (86%)**, followed by addition errors (10.5%) and incorrect facts (3.2%). **Accuracy was inversely correlated with transcript length and data complexity**.
  - The **quality of AI-generated notes, assessed by PDQI-9, varied substantially**, with a mean score of **29.7 (range 23.7-39.7)**, closer to the "terrible or bad" range in previous research. This variability makes it "difficult for health care providers to predict where errors may occur".
  - **Hallucinations** (false information produced by the AI) are a marked concern. In one instance, an AI scribe summarized that a prostate examination had been performed when the physician only mentioned scheduling it. Another example saw "issues with the patient's hands, feet, and mouth" being summarized as a diagnosis of "hand, foot, and mouth disease".
  - The Sheffield Assessment Instrument for Letters (SAIL) showed **AI tool-assisted notes and letters had higher scores** (70% vs 29% for letters, 100% vs 43% for notes, scoring >25) compared to standard EHR, indicating **improved completeness and content**.
- **Impact on Patient Engagement and Experience**
  - AI-facilitated documentation can lead to **more engaged and personable provider-patient encounters**, as clinicians spend less time focused on the computer. Physicians reported an increase in the proportion of clinicians agreeing they could "give my patients my undivided attention" from **57.9% to 93.0%**.
  - Early patient feedback has been positive, with some describing **improved interaction** with their physicians. The median provider-specific **likelihood to recommend increased from 92.3% to 94.3%** after digital scribe implementation.
  - However, some patients might feel uncomfortable with the recording or AI use, and transparent patient consent processes are necessary.
- **Role of Large Language Models (LLMs) and Multimodal LLMs (MLLMs)**
  - LLMs, such as **GPT-4**, are central to many AI scribe tools, converting consultation audio into medical notes.
  - MLLMs, which **integrate multiple data modalities beyond text** (e.g., images, audio, time-series, sensor inputs), are rapidly emerging in healthcare. Most included MLLM studies (81%) examined applications in **diagnosis and reporting** in fields like radiology, pathology, and ophthalmology.
  - Their potential extends to medical education, surgical assistance, public mental health assessment, and security vulnerability evaluation.
  - While MLLMs offer "transformative capabilities" by dynamically updating patient records in real time from multimodal data streams, issues like **"hallucinations"** (false information produced by the AI) need to be addressed. A pre-study development phase of one AI tool identified a possible **major hallucination rate of 0.21%** in dummy notes, which was later reduced to 0% with post-study development efforts.
- **Integration Challenges and the Need for Human Oversight**
  - Despite efficiency gains, AI tools often require **manual review and editing** by clinicians. This "can sometimes offset the time saved".
  - One study noted that clinicians often still needed to add sections to the notes manually to account for **specialty-specific needs**, which could increase total note length and time spent.
  - Full integration into Electronic Health Record (EHR) systems is crucial. Although ambient AI tools were found to reduce burnout despite requiring clinicians to work with a mobile device and a separate website, **comprehensive EHR integration** is still seen as a future need.
  - The human element remains crucial for oversight, especially for complex cases and ethical considerations. Clinicians reported having **initial "trust issues"** with the technology, though confidence improved over time.
- **Ethical and Regulatory Considerations**
  - Concerns exist regarding **patient privacy, data governance, and potential biases** in AI models. For example, AI models could "imagine or misgender patients" or offer "unsolicited and inappropriate diagnoses".
  - Regulatory frameworks and ethical standards are crucial. The application of AI in healthcare, if its intended use becomes a medical purpose, means it **becomes subject to Medical Device Regulation (MDR)**.
  - Research also highlights the need to address **algorithmic bias**, particularly in under-represented populations, through inclusive model development and continuous monitoring.

#### Opportunities and Gaps for Future Research

The sources consistently point to several areas requiring further investigation:

- **Further Evaluation of Effectiveness and Impact**:
  - Conduct **larger cohort, longitudinal studies** across diverse clinical settings, patient populations, and specialties (including urgent care, emergency department, inpatient settings) to assess long-term impacts on documentation time, cognitive load, burnout, productivity, and patient outcomes.
  - Investigate the impact of AI scribes on broader functions of clinical notes, such as **medical synthesis, clinical communication, and clinical reasoning**, beyond mere note creation.
  - Explore how AI impacts functions like **order entry or patient instructions**.
  - Evaluate the impact of more advanced technologies (e.g., DAX Copilot's advanced versions).
- **Improving Accuracy and Reliability**:
  - Address the challenges of **hallucinations and inaccuracies** by developing refined AI tools that consistently meet high clinical standards.
  - Focus on **real-time error correction** and improved recognition capabilities, especially for **accented or non-native speech**.
  - Establish **standardized evaluation metrics and structured error analysis protocols** for MLLMs to allow for fair comparisons across models, tasks, and use cases, ensuring robustness and clinical meaningfulness.
- **Enhancing User Experience and Workflow Integration**:
  - Develop more customizable AI tools that adapt to **individual clinician styles, departmental needs, and note formatting preferences**.
  - Focus on **seamless EHR integration** to eliminate barriers and blend documentation smoothly into existing workflows.
  - Investigate effective **training programs** and ongoing support mechanisms for clinicians adopting AI technologies.
  - Research the impact of **task switching reduction** as a major contributor to time saved.
- **Addressing Ethical, Legal, and Privacy Concerns**:
  - Prioritize research on transparent **data governance, secure data management, and robust anonymization practices**.
  - Explore and address issues of **algorithmic bias** to ensure equitable care, particularly when AI is trained on limited or biased datasets.
  - Conduct studies on **patient perspectives and comfort** with AI use, including informed consent processes, and how AI impacts the patient-physician relationship.
- **Cost-Effectiveness and Return on Investment (ROI)**:
  - Perform comprehensive **cost-benefit analyses** and assess the ROI of AI scribe implementation to justify widespread adoption, considering factors like improved physician productivity and retention.
- **Expanding AI Capabilities and Applications**:
  - Further develop **multimodal LLMs** to integrate diverse data sources (e.g., audio, time-series, sensor inputs) for enhanced diagnostic accuracy and contextual understanding.
  - Investigate **underexplored application areas** beyond current diagnostic and documentation uses, such as surgical assistance, public mental health assessment, and security vulnerability evaluation.
  - Research ways to provide AI feedback on human clinical reasoning, especially for educational purposes.
- **Methodological Improvements**:
  - Future reviews should consider **stricter inclusion criteria** and adherence to established reporting guidelines to enhance comparability and validity in a rapidly evolving field.
  - Address the issue of **reproducibility** in LLM outputs, ensuring consistent results under varied conditions.
  - Incorporate **human-centered design practices**, emphasizing usability, interpretability, and clinician involvement.
  - Develop **inclusive datasets** that reflect real-world demographic and clinical diversity for better generalizability.

### **Follow-up Prompt 2**

Based on the provided articles, add to this list of opportunities any information about potential for more evaluation methods, applications in various specialties or clinical disciplines, and medical educational considerations or impacts

### **Follow-up Response 2**

The provided articles extensively highlight various opportunities for the future development and application of AI in healthcare documentation, extending beyond current capabilities into new evaluation methods, diverse clinical applications, and significant educational impacts.

Here's an expanded look at these opportunities, supported by specific findings:

#### Potential for More Evaluation Methods

Future research needs to address the methodological inconsistencies and limitations observed in current evaluations to establish more robust evidence for AI scribe effectiveness and safety.

- **Standardized Metrics and Structured Error Analysis:**
  - The **heterogeneity of methods utilized to assess document quality** currently influences the interpretation of results. There is a critical need for **standardized evaluation metrics and structured error analysis protocols** to allow for fair comparisons across models, tasks, and use cases.
  - While **accuracy** is the most commonly reported metric (67% of MLLM studies), metrics like **precision, recall, and F1-scores** (used in 26% of studies) are underused, despite their importance for classification tasks. Similarly, **diagnostic metrics like AUROC** (used by only 8% of studies) are critical for clinical risk stratification but are not widely applied.
  - **Error analysis** currently lacks depth and structure, with **77% of studies offering only partial or example-based analyses** without systematic categorization or frequency quantification. Only **18% of MLLM studies conducted systematic error analysis**, distinguishing failure types like hallucinations or reasoning errors. This highlights an urgent need for **task-specific and standardized error evaluation frameworks**.
  - New standards should define **acceptable error thresholds and mechanisms for corrective actions** in AI-generated documentation.
- **Broader Study Designs and Data Collection:**
  - **Larger cohort, longitudinal studies** are needed across diverse clinical settings and patient populations to assess long-term impacts on documentation time, cognitive load, burnout, productivity, and patient outcomes. Current studies are often short-term pilots with small sample sizes, limiting generalizability.
  - **Randomized controlled designs** are recommended to validate findings across varied healthcare settings and populations.
  - Include **structural, process, and patient outcomes** in future work, as current studies often rely on clinician perceptions rather than direct patient experience or broader system impacts. **Patient survey data** should be incorporated to understand patient opinions and fears regarding new technology.
  - Investigate the **reproducibility of LLM outputs** under varied conditions to understand reliability.
  - Conduct **formal audits of the quality and usability of AI-generated notes** to enhance future study of this technology.
  - A **dynamic evaluation framework** is essential given the rapid pace of technological change, assessing performance across domains including engagement, effectiveness, quality, and safety. This includes **ongoing benchmarking, evaluation, and monitoring**.
- **Cost-Effectiveness and Return on Investment (ROI):**
  - Comprehensive **cost-benefit analyses** and assessment of ROI are crucial to justify widespread adoption, considering factors like improved physician productivity and retention. Current findings on cost-effectiveness are inconclusive.

#### Applications in Various Specialties or Clinical Disciplines

AI's potential extends far beyond its current primary use in general outpatient documentation, with opportunities for specialized applications and integration across numerous medical fields.

- **Expanding Beyond Primary and Ambulatory Care:**
  - While many studies focus on primary care and various ambulatory specialties (e.g., pediatric cardiology, endocrinology, gastroenterology, dermatology, orthopedics, otolaryngology, neurology, neurosurgery, psychiatry, sports medicine, immunology, rheumatology), further studies should evaluate use in **urgent care settings, emergency departments, and inpatient settings**.
  - **Nursing documentation** is a significant area where multimodal LLMs (MLLMs) can reduce administrative burdens, enhance care personalization, and improve nurse-patient interactions by integrating audio, video, and text data in real-time.
  - MLLMs are demonstrating capabilities in **diagnosis and reporting across diverse medical domains**, including radiology, pathology, and ophthalmology. Specific applications include:
    - **Dermatological diagnosis** by combining skin images with clinical concepts.
    - **Radiology report generation and image interpretation** (e.g., chest X-rays for pneumonia, brain MRIs for neurological conditions, CT scans for organ damage).
    - **Pathology** for automated histopathological and cytopathological analysis for cancer detection and disease classification.
    - **Cardiology**, specifically ECG interpretation and identification of conditions like myocardial infarctions and arrhythmias.
    - **Prostate cancer detection** using 3D MRI scans and integrating clinical guidelines.
    - **Automated radiotherapy treatment planning** by streamlining tumor segmentation, dose optimization, and beam arrangement.
- **Beyond Documentation:**
  - MLLMs have the potential for applications in **surgical assistance**, **public mental health assessment**, and **security vulnerability evaluation** (e.g., differentiating fake from real images to avoid fraud reports).
  - AI can facilitate **insurance prior authorization** by compiling necessary patient record elements and automating review processes.
  - AI can help **decrease barriers to healthcare access**, offering remote consultations and access to educational resources, particularly in underserved areas. It can also streamline **transfer of care** to reduce medical errors during handoffs.

#### Medical Educational Considerations or Impacts

AI in healthcare documentation has direct and indirect implications for medical education, offering new tools for learning, feedback, and skill development, while also necessitating curricular changes to prepare future clinicians.

- **Enhancing Clinical Reasoning (CR) and Feedback:**
  - AI holds promise to fill the gap in **limited feedback provided to physicians on clinical reasoning**. Tools can provide more granular feedback across domains like differential diagnosis and explanation of reasoning.
  - The use of LLM-based assessments of CR documentation is a **multi-institutional approach** that enhances generalizability and robustness for educational purposes. AI models can perform classification tasks for CR documentation quality with high accuracy (F1-score >0.70, AUROC >0.70 acceptable for low stakes formative feedback).
  - Future steps include integrating these AI-based assessments into residency programs to provide **formative feedback**, including dashboards to visualize longitudinal trends and set improvement goals for residents. This supports the essential human skill of CR, as AI is **not yet performant enough to replace human reasoning**.
- **Patient Education and Engagement:**
  - LLMs can increase **patient engagement** by converting complex medical information into an understandable format at a target reading grade level of the patient’s choosing. They can provide **personalized education** on disease, treatment options, and self-care advice.
  - AI can facilitate **patient-centered communication**, allowing clinicians to spend less time typing and more time engaging with patients. Patients have reported improved interaction with their physicians.
- **Curriculum and Training:**
  - The **central and pivotal role of physicians in advancing EHR AI** should be a focus in undergraduate and professional training in medical school curricula. This includes educating on the **appropriate and inappropriate uses of medical scribes**.
  - **AI literacy and capacity building** are needed from the bedside to top leadership around the appropriate procurement, implementation, and use of AI technologies.
  - Training material must **reinforce the ongoing responsibility of physicians to review and revise all clinical documentation**, whether or not assisted by AI. This is critical for safety and accuracy given the potential for **hallucinations and inaccuracies** in AI-generated content.
  - LLMs can encode clinical knowledge. Studies have assessed LLM performance on medical licensing exams (e.g., USMLE, Japanese Medical Licensing Exam), showing potential for **AI-assisted medical education** and diagnostic support.
  - AI can be leveraged to assist in **qualitative thematic analysis of interviews**, potentially expediting research in medical education and healthcare systems.

These opportunities underscore that the role of AI in healthcare is rapidly expanding beyond mere documentation, requiring continuous development, rigorous evaluation, and thoughtful integration into both clinical practice and medical education.
