# Supplementary figures and images for: AI Scribes in Health Care: Balancing Transformative Potential With Responsible Integration
Source: JMIR Med Inform. 2025 Aug 1;13:e80898. doi: 10.2196/80898 (PMC12316405; doi:10.2196/80898)

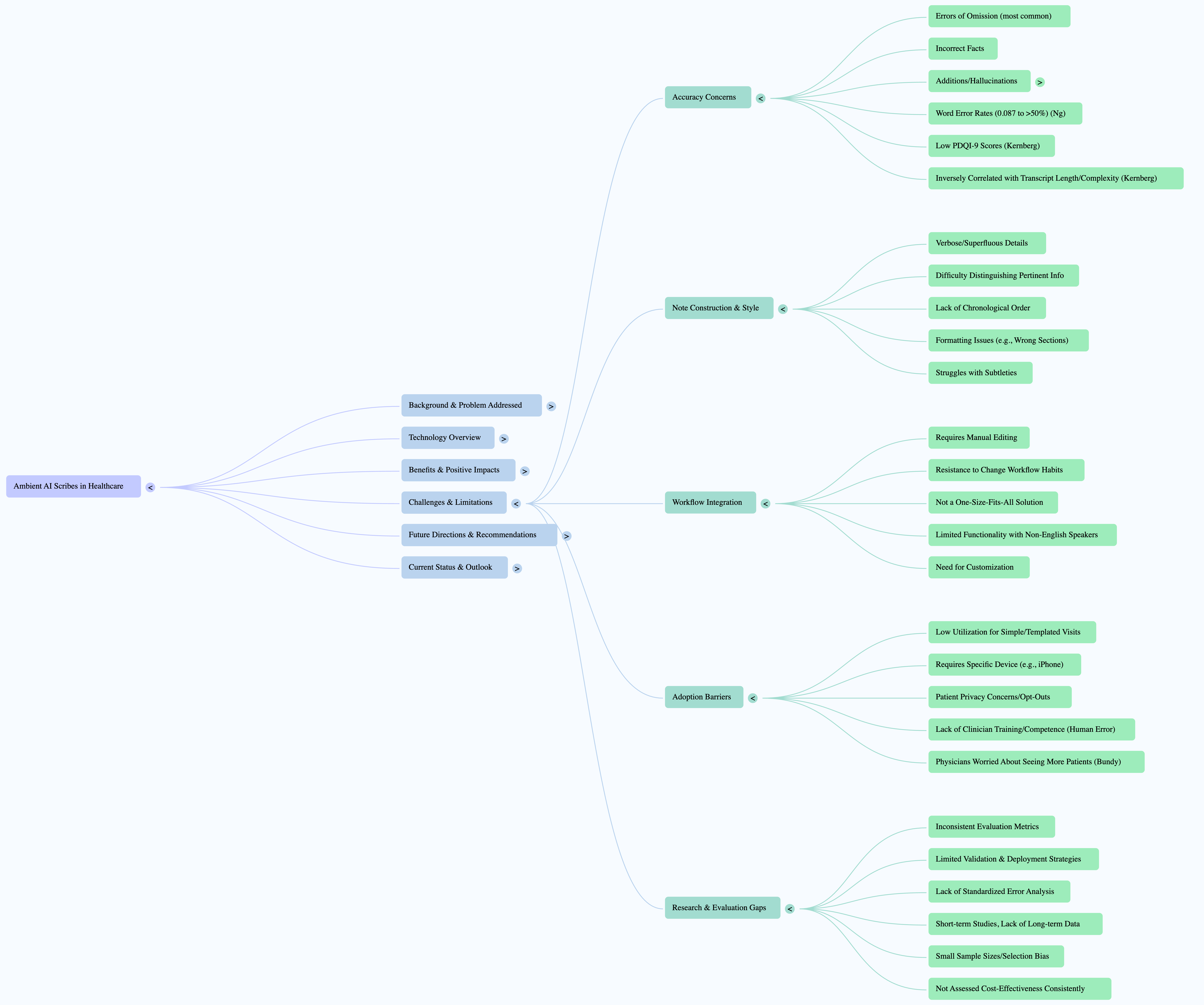

Supplement: Multimedia Appendix 3 [file medinform-v13-e80898-s003.png]

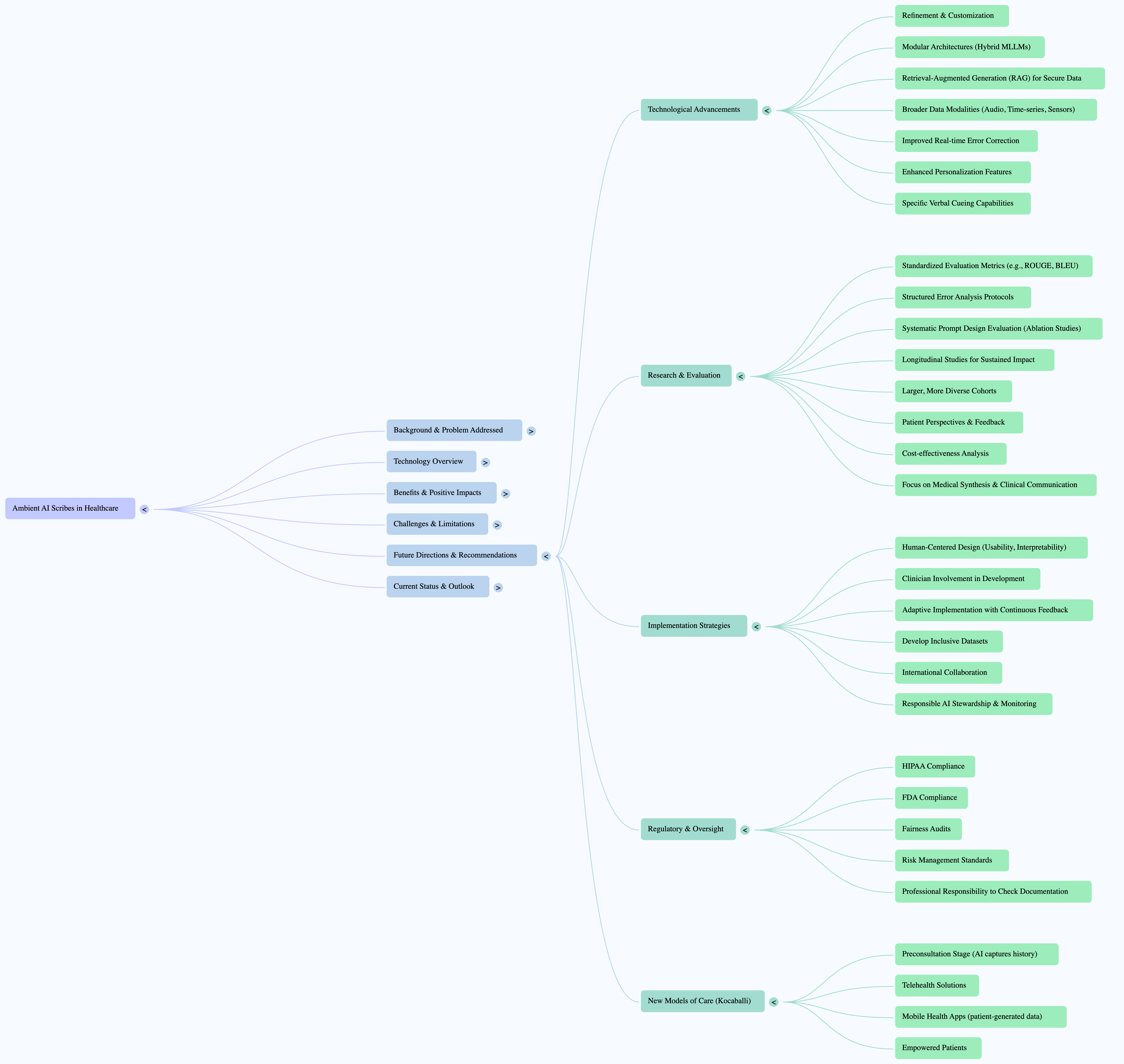

Supplement: Multimedia Appendix 4 [file medinform-v13-e80898-s004.png]
